# Diffusion tensor brain imaging at 0.55T: A feasibility study

**DOI:** 10.1002/mrm.30156

**Published:** 2024-05-09

**Authors:** Hao-Ting Kung, Sophia X. Cui, Jonas T. Kaplan, Anand A. Joshi, Richard M. Leahy, Krishna S. Nayak, Justin P. Haldar

**Affiliations:** 1 Signal and Image Processing Institute, Ming Hsieh Department of Electrical and Computer Engineering, University of Southern California, Los Angeles, California USA; 2 Siemens Medical Solutions USA, Los Angeles, California USA; 3 Brain and Creativity Institute, University of Southern California, Los Angeles, California USA

**Keywords:** constrained reconstruction, denoising, diffusion MRI, low-field MRI

## Abstract

**Purpose:**

To investigate the feasibility of diffusion tensor brain imaging at 0.55T with comparisons against 3T.

**Methods:**

Diffusion tensor imaging data with 2 mm isotropic resolution was acquired on a cohort of five healthy subjects using both 0.55T and 3T scanners. The signal-to-noise ratio (SNR) of the 0.55T data was improved using a previous SNR-enhancing joint reconstruction method that jointly reconstructs the entire set of diffusion weighted images from k-space using shared-edge constraints. Quantitative diffusion tensor parameters were estimated and compared across field strengths. We also performed a test–retest assessment of repeatability at each field strength.

**Results:**

After applying SNR-enhancing joint reconstruction, the diffusion tensor parameters obtained from 0.55T data were strongly correlated (*R*^2^ ≥ 0.70) with those obtained from 3T data. Test–retest analysis showed that SNR-enhancing reconstruction improved the repeatability of the 0.55T diffusion tensor parameters.

**Conclusion:**

High-resolution in vivo diffusion MRI of the human brain is feasible at 0.55T when appropriate noise-mitigation strategies are applied.

## 1 | INTRODUCTION

There has recently been a substantial resurgence of interest in MRI systems with lower B_0_ field strengths.^1–7^ These systems can now perform much better than historical lower-field magnets due to modern improvements in MRI hardware (e.g., magnet design, gradient systems, etc.), and offer potential advantages in value and accessibility (among other possible benefits) compared to higher-field systems. However, the reduced signal-to-noise ratio (SNR) associated with lower B_0_ is a major potential concern. SNR limitations are especially problematic for applications such as quantitative diffusion MRI, which can provide powerful insights into tissue microstructure and anatomical connectivity,^8–10^ but where noise can already be worrisome even at higher field strengths.

In this study, we investigated the basic feasibility and baseline characteristics of in vivo diffusion tensor brain imaging on a prototype 0.55T MRI system in a small cohort of healthy volunteers. Our study design enables a novel comparison between quantitative diffusion metrics obtained at 0.55T against results obtained from the same subjects on a conventional 3T system, and includes an assessment of repeatability at each field strength. Compared to existing work, our 0.55T acquisition had substantially higher spatial resolution than was used in previous low-field diffusion MRI studies.^2,11–20^ To mitigate the SNR concerns that arise with higher-resolution acquisitions, we used a general and theoretically well-characterized SNR-enhancing (denoising) reconstruction approach that has previously demonstrated high efficacy in various human and animal diffusion MRI applications across a range of resolutions and field strengths.^21–23^

Our results suggest that when suitable noise-mitigation methods are used with the 0.55T data, it is possible to obtain strong correlation between diffusion tensor metrics obtained at 0.55 and 3T, and that diffusion tensor metrics obtained at 0.55 and 3T can have similar levels of repeatability. Preliminary accounts of portions of this work were previously presented at recent conferences.^24,25^

## 2 | METHODS

### 2.1 | Data acquisition

MRI data was collected from five healthy volunteers, who were each scanned twice using a whole-body 0.55T system (prototype MAGNETOM Aera, Siemens Healthineers) equipped with high-performance shielded gradients (45 mT/m amplitude, 200 T/m/s slew rate). The two 0.55T scans for each subject were performed on the same day, although subjects exited and reentered the magnet in between repetitions. The same subjects were also scanned twice on the same day using a conventional 3T system (MAGNETOM Prisma^fit^, Siemens Healthineers), where the 3T scans took place either on the same day (one subject), one day before (two subjects), or one day after (two subjects) each subjects’ corresponding 0.55T scans. All MRI experiments were performed with approval from the Institutional Review Board of the University of Southern California.

Our scan protocol for both field strengths involved the collection of T1-weighted MPRAGE anatomical reference images with 1 mm isotropic resolution (with matched acquisition times between field strengths) as needed for automated processing steps (e.g., image registration, brain extraction, segmentation, etc.), a B_0_ field map to enable correction of susceptibility-induced distortions, and EPI-based single-shell diffusion MRI data (64 directions with *b* = 1000 s/mm^2^ and one image with *b* = 0 s/mm^2^).

The diffusion acquisitions were largely similar between the 0.55T and 3T, with matched FOV (260 mm × 260 mm) and matrix size (130 × 130), slice thickness (2 mm thickness with 0% distance factor), repetition time (TR = 10000 ms), strong fat saturation, scan duration (11 min and 2 s), and 6/8ths partial Fourier acquisition along the phase encoding dimension (anterior–posterior) with no parallel imaging or multiband acceleration. There were also some differences in the acquisition protocol, which arose primarily because the 3T scanner had a more powerful gradient system (80 mT/m amplitude, 200 T/m/s slew rate). This led to differences in the echo time (TE = 90 ms for 0.55T; TE = 80 ms for 3T), total number of slices (56 for 0.55T; 60 for 3T), echo spacing (0.79 ms for 0.55T; 0.58 ms for 3T), bandwidth (1374 Hz/Px for 0.55T; 1924 Hz/Px for 3T), and diffusion encoding scheme (monopolar for 0.55T; bipolar for 3T). Due to differences in the 1H resonance frequency, the two systems also necessarily used different receiver coils. The 0.55T data was acquired using a 16-channel head and neck receiver array, while the 3T data was acquired using a 20-channel head and neck receiver array.

Our choice to acquire 0.55T diffusion MRI data at high resolution (nominally 2 mm isotropic resolution, corresponding to an 8 mm^3^ voxel volume) is perhaps atypical, since SNR is expected to be especially limited at this spatial resolution and field strength. This decision was guided by our previous theoretical observation^26^ that, when denoising methods will be employed, it can be substantially more efficient to acquire noisy data at higher-than-nominal spatial resolution instead of acquiring higher-SNR data exactly at the nominal resolution (Also see Reference 27 for a discussion of early theoretical misconceptions about the trade-off between spatial resolution and SNR in MRI, which may have contributed to a historical underappreciation of the potential advantages of denoising high-resolution noisy data.).

### 2.2 | Image reconstruction

Diffusion-weighted images were reconstructed from raw k-space data using in-house software. We implemented two different types of reconstruction for the 0.55 T diffusion data:

1. An SNR-enhancing joint reconstruction (SER) that integrates image formation from multichannel k-space data, denoising, partial Fourier resolution recovery, and Gibbs ringing mitigation all in a single step.^21–23^ This type of single-step k-space-based approach is generally more difficult to implement and less accessible than common multistep image-domain approaches. However, single-step approaches enable the maximum possible utilization of information from the original data compared to multistep methods that typically lose useful information in each subsequent processing step (see, e.g., the data processing inequality from information theory^28^), which can degrade overall performance.^29^ Our formulation included SENSE-based^30^ maximum-likelihood modeling of the multichannel k-space data with sensitivity maps estimated using PISCO^31^; regularization (using an antilinear operator-based implementation^32^) of the imaginary parts of phase-compensated images to enable partial Fourier recovery^21,33^; and the use of a compound Markov random field regularization penalty that jointly imposes smoothness constraints on all of the diffusion weighted images (for the purposes of SNR-enhancement and Gibbs ringing mitigation) while also estimating the joint edge structure shared by all of the images to avoid the blurring of information across image edges.^21^ This approach has the advantage that the amount of SNR-enhancement and spatial resolution degradation will be fairly consistent between diffusion images, and is easily quantified such that regularization parameters can be tuned to achieve an appropriate balance between SNR and spatial resolution that ensures good SNR efficiency.^26^ In addition, this formulation can avoid the interimage signal leakage that often occurs when multiple images are processed using advanced denoising or reconstruction methods that take advantage of the shared features of a multicontrast image sequence.^34^ Attaining consistent spatial resolution for all of the diffusion-weighted images and avoiding interimage signal leakage can be important for ensuring the interpretability of later quantitative parameter estimation steps.^21^
2. For comparison, we also performed a conventional noniterative Fourier reconstruction of the data from each coil (zero-filling the missing partial Fourier information and using a hamming window to mitigate Gibbs ringing), followed by SENSE-based coil combination using the same sensitivity maps from before.

The spatial resolution for both reconstructions was measured by calculating the full-area at half-maximum of the two-dimensional spatial-response function (analogous to the conventional full-width at half-maximum that is frequently used to quantify one-dimensional resolution).^26^ The spatial resolution was anisotropic because of the partial Fourier acquisition, but the effective in-plane resolution was (2.61 mm)^2^ for the conventional reconstruction. The SER parameters were adjusted to achieve a roughly 4× SNR improvement in smooth parts of the image (the SNR improvement was less in the vicinity of image edge structures^21^), with a corresponding effective in-plane resolution of (3.15 mm)^2^ (with better resolution than this in the vicinity of image edge structures). Note that this loss of spatial resolution is quite modest compared to the very substantial improvement in SNR, and also avoids the exacerbation of partial volume artifacts that would occur if smoothing across high-resolution edge features.

To avoid EPI Nyquist ghosts, we estimated linear phase correction factors prior to image reconstruction to account for the mismatch between the even and odd lines of k-space data. This was achieved by using variable projection methods^35^ to minimize a cost function that combined a least-squares penalty on the mismatch between even and odd lines in phase-corrected three-line EPI navigator data^36^ and a nonconvex penalty that encourages low-rank structure in a Toeplitz matrix (corresponding to the suppression of Nyquist ghosts^37^) formed from the phase-corrected k-space data of interest for each image.

For comparison, we also reconstructed the 3T diffusion images from raw k-space data to serve as a reference for the0.55Tresults.However,althoughthe3Tdataislikelyto also benefit from SNR-enhancing reconstruction,^21,23^ we only applied the conventional reconstruction approach to the 3T data to avoid the possibility of having a comparison reference that is unfairly biased toward SER. This choice means that if any bias exists in our 0.55 versus 3T comparison, this bias should make the 0.55T conventional reconstruction appear better than it is and make the 0.55T SER approach appear worse than it is. This ensures that if we observe that SER has an advantage over conventional reconstruction in the 0.55 versus 3T comparison, then this is likely to be a true advantage.

### 2.3 | Postprocessing and parameter estimation

Following reconstruction, we used a fieldmap-based distortion correction technique from the BrainSuite Diffusion Pipeline^38^ to compensate for susceptibility-induced geometric distortions. (Note that, due to phase wrapping issues that occured in our 0.55T fieldmap acquisition, it was necessary to correct the fieldmap using a phase unwrapping approach^39^). Gradient nonlinearity correction was then applied to both the diffusion results and the T1-weighted anatomical image using software from the Human Connectome Project’s preprocessing pipeline^40^ together with spherical harmonic coefficients describing each gradient system. Diffusion tensors were then using the BrainSuite Diffusion Pipeline.^38^ From the estimated tensors, we extracted the orientation of the primary eigenvector for each voxel, and calculated quantitative parameters like mean diffusivity (MD) and fractional anisotropy (FA). The diffusion-weighted images were rigidly coregistered to the corresponding T1-weighted anatomical image.^41^ Finally, we used FSL to rigidly align the 0.55 and 3T anatomical scans from the same subject,^42^ and applied corresponding spatial transformations to align the diffusion tensors.^43^

### 2.4 | Analysis

We performed several statistical analyses to assess the degree of consistency between the 0.55T results and the 3T results.

First, we used a simple linear regression model to understand the degree to which the 0.55T MD and FA valuescouldbepredictedbythecorresponding3TMDandFA values from the same spatial location in the same subject. To understand the importance of noise mitigation, this linear regression was performed for both the SER and the conventional reconstruction of the 0.55T data. This analysis was performed only for spatial locations within the cerebrum (excluding the cerebellum, brainstem, and nonbrain tissues), while excluding regions in the frontal and temporal lobes where substantial susceptibility-induced geometric distortion were evident in the 3T data, and also excluding spatial regions that did not appear in all of a subjects’ scans due to slight variations in the position of the field-of-view. Goodness of fit for the regression models was assessed using the coefficient of determination (*R*^2^), and we follow the somewhat arbitrary convention that an *R*^2^ value greater than or equal to 0.70 (i.e., indicating that 70% or more of the variance in the dependent variable is explained by the regression model) as indicative of a strong correlation.

We also analyzed how well the tensor orientations matched between 0.55 and 3T. Specifically, we calculated the angular discrepancy between orientations estimated with 0.55 and 3T data at the same spatial location in the same subject using the arc-cosine of the dot-product between the orientations of the primary eigenvectors of each diffusion tensor. This analysis was restricted to white matter voxels (as identified by BrainSuite’s segmentation of the 3T anatomical image) where the 3T FA value was *>*0.3 (the choice of 0.3 as a threshold is not uncommon, though somewhat arbitrary), but otherwise used the same sets of voxels that were used in the regression analysis.

We also used Bland–Altman plots^44^ to evaluate the test-retest repeatability of MD and FA values at both 0.55 and 3T, using the same sets of voxels used in the regression analysis. As is standard in Bland–Altman analysis, we calculated the bias (i.e., the mean of the difference) between the two measurements as well as the 95% limits of agreement (i.e., the interval within which approximately 95% of all test–retest differences would lie, assuming a normal distribution). To provide further insight into repeatability, we also calculated intra-subject coefficients of variation.

## 3 | RESULTS

**FIGURE 1:**
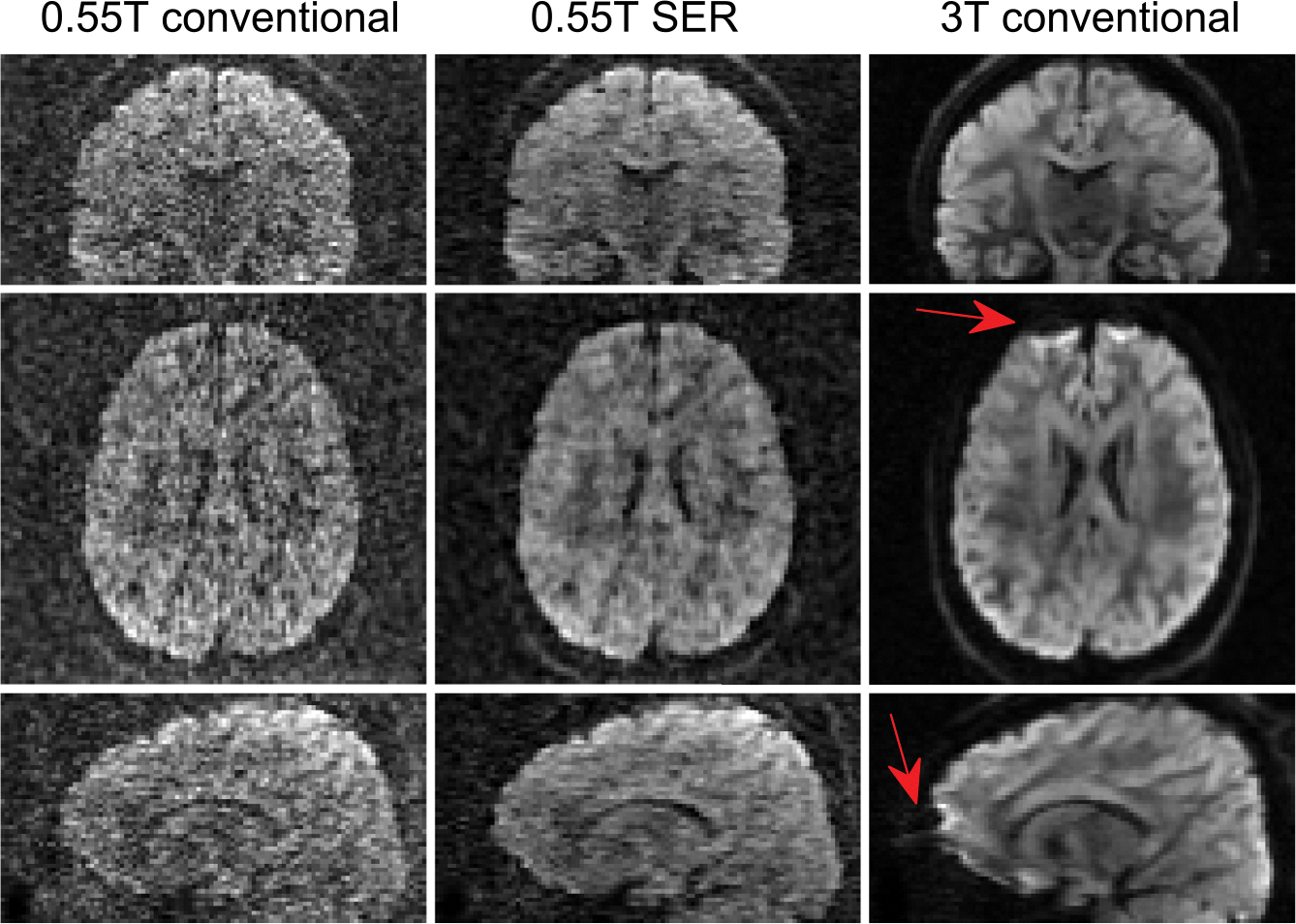
Representative (top) coronal, (middle) axial, and (bottom) sagittal slices from a diffusion-weighted imaging obtained using (left) conventional reconstruction applied to 0.55T data, (middle) signal-to-noise ratio-enhancing joint reconstruction applied to the same 0.55T data, and (right) conventional reconstruction applied to 3T data. Locations where incompletely resolved geometric distortion artifacts are present in the 3T data are indicated with arrows. Note that we tried to choose similar slices for 0.55 and 3T, but these images are not perfectly aligned.

**FIGURE 2:**
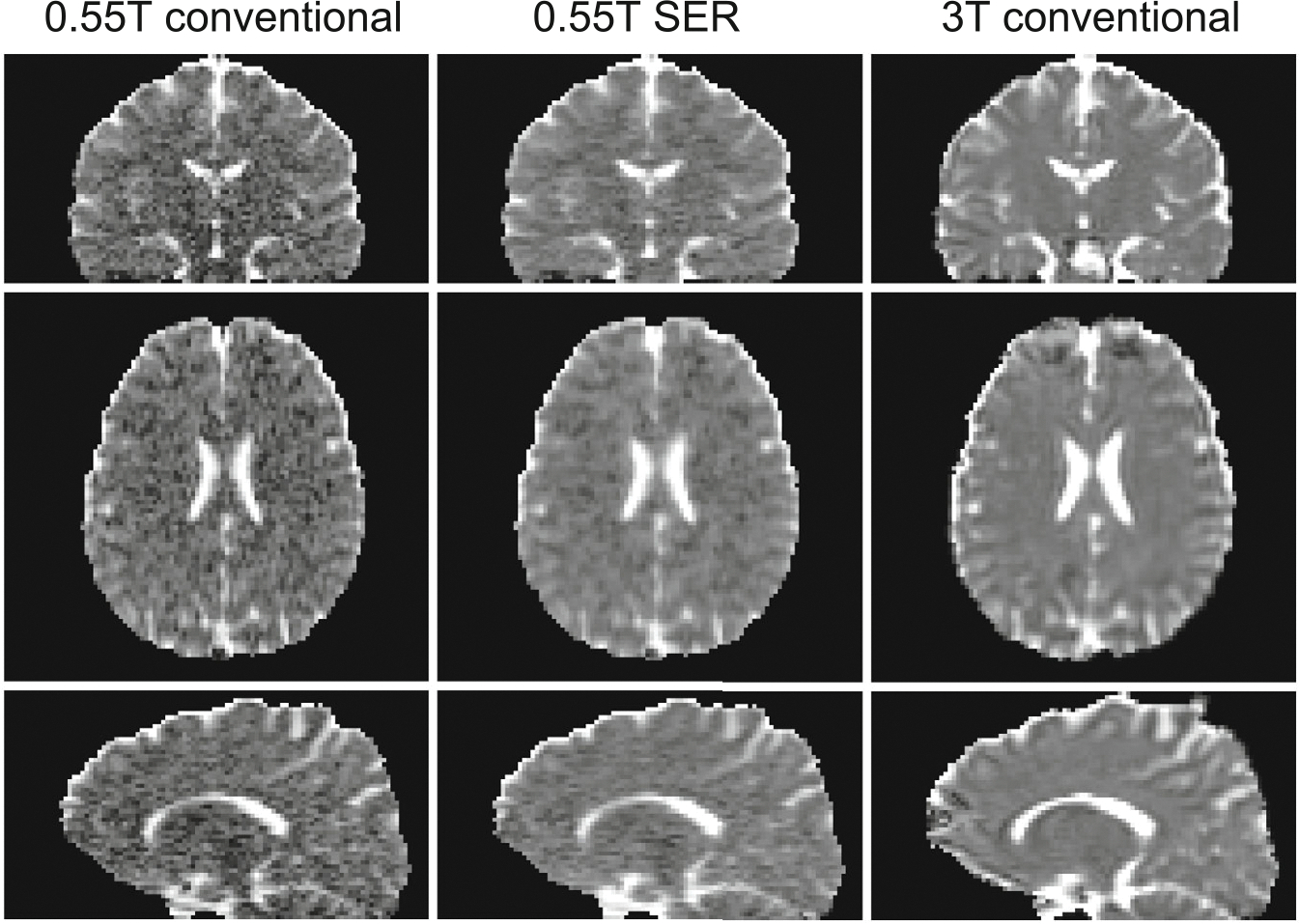
Maps of mean diffusivity (MD) corresponding to the same cases depicted in Figure 1.

**FIGURE 3:**
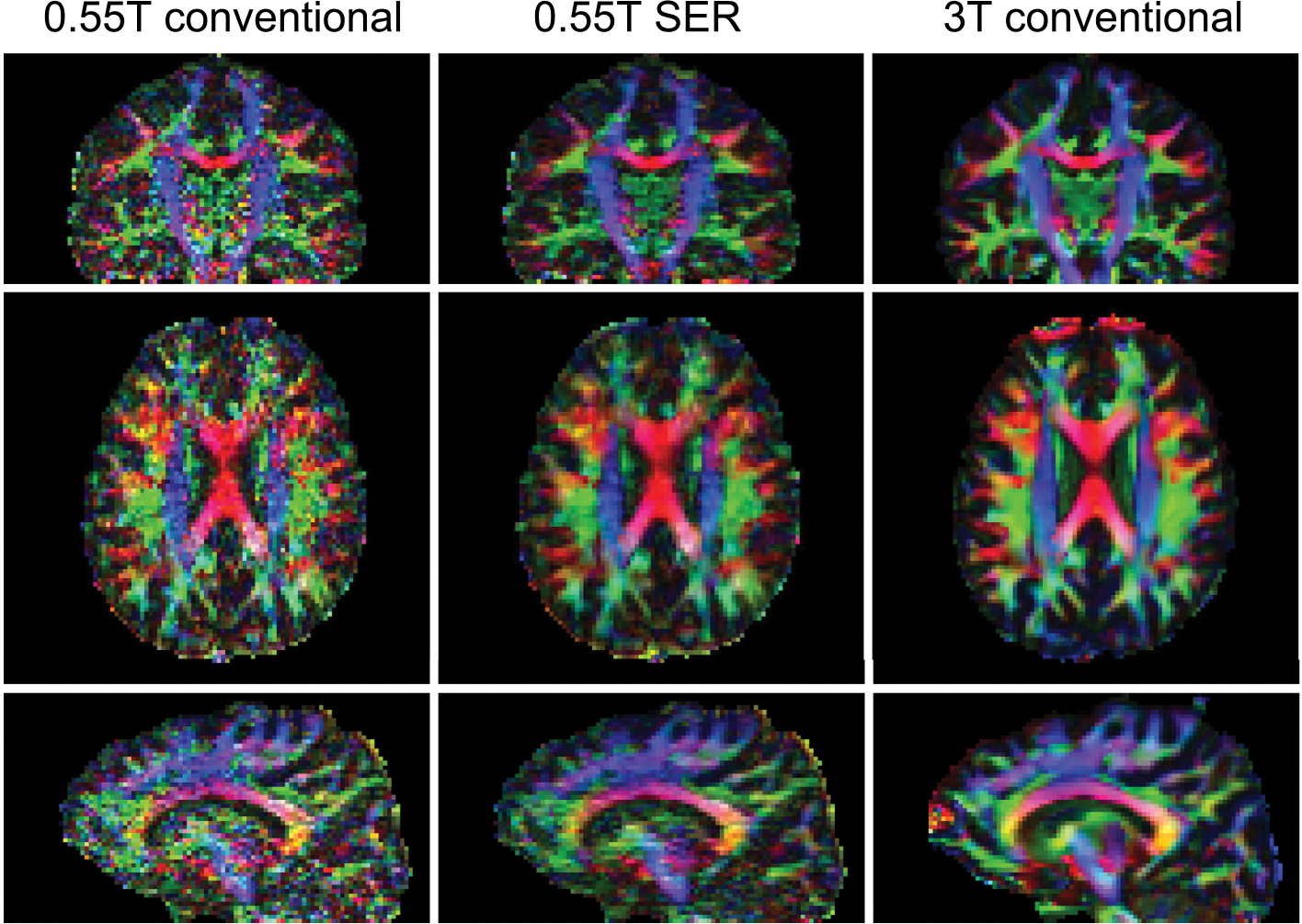
Color-coded fractional anisotropy (FA) maps corresponding to the same cases depicted in Figures 1 and 2.

Qualitative visual illustrations of the reconstructed DWIs, and the corresponding MD and FA maps are shown in Figures 1, 2, 3, respectively. We observe that DWIs obtained with conventional reconstruction of the 0.55T data are visually quite noisy, while the SER DWIs are visually less-noisy, as expected. The DWIs obtained with conventional reconstruction of the 3T data visually appear to have the highest SNR, also as expected. However, it is also visually noticeable (e.g., see the frontal lobe) that the 3T images have stronger incompletely resolved artifacts related to susceptibility-induced geometric distortion than the 0.55T data, which is consistent with the physics of acquisition at 3T.

**FIGURE 4:**
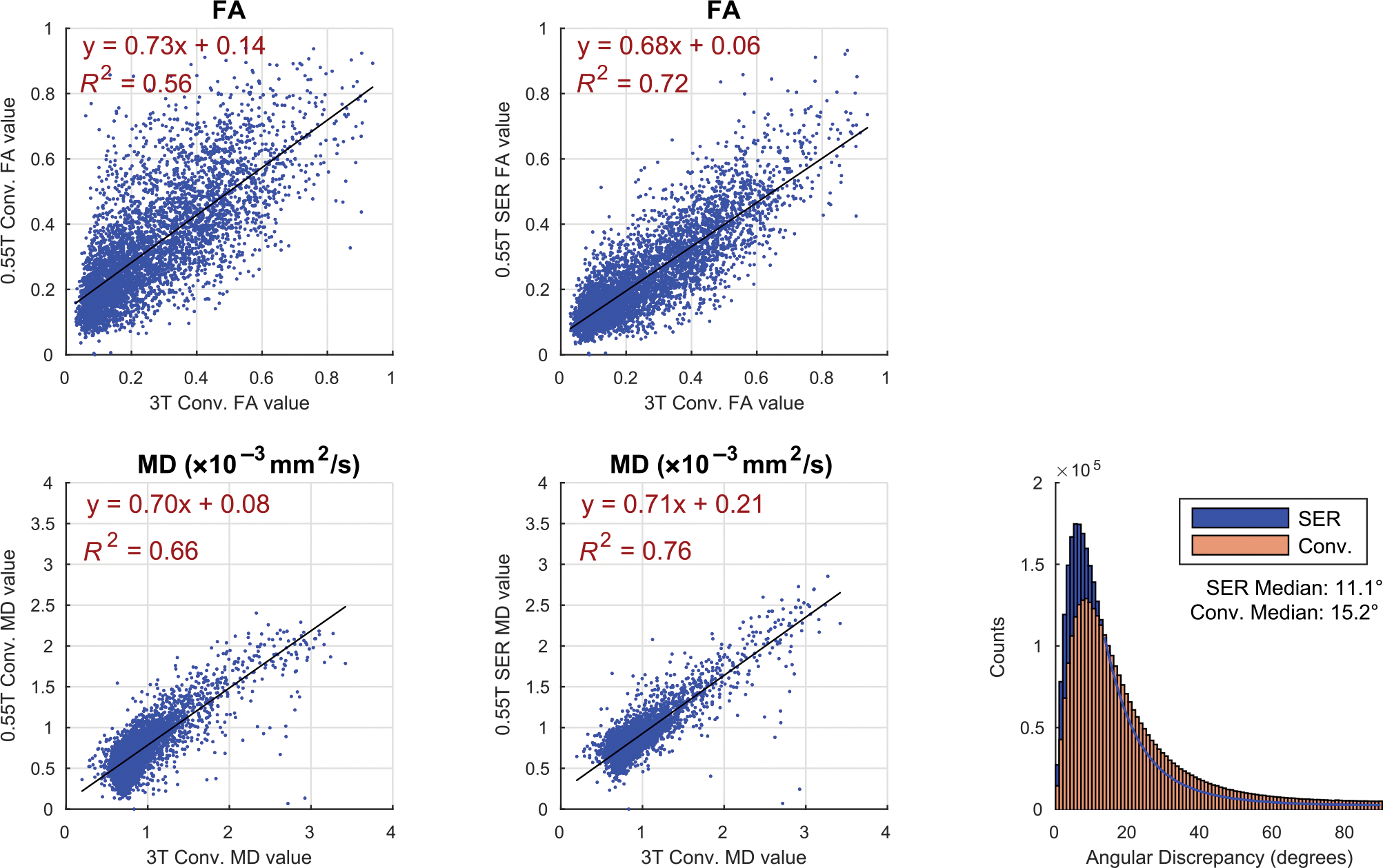
(Left) Linear regression analyses and (right) angular discrepancy analyses. In the linear regression analyses, we use scatter plots to show the correspondence between values obtained at 0.55T and values obtained at 3T for both fractional anisotropy (FA) and mean diffusivity (MD) and for both 0.55T reconstruction methods, and also show the corresponding best fit regression lines and the *R*^2^ values. For visualization purposes, we are only showing 0.05% of the samples (randomly selected), but the regression is calculated based on all of the samples. For the orientation analyses, we show histograms of the angular discrepancy between 0.55 and 3T for the two different 0.55T reconstruction approaches. In these plots, “conv.” stands for “conventional.”

Figure 4 shows the results of our linear regression and angular discrepancy analyses. As can be seen, the FA and MD values obtained with both reconstructions of the 0.55T data were well-correlated with the corresponding 3T values (*R*^2^ ≥ 0.50 in all cases). However, the SER-based 0.55T values had higher correlation with 3T than was observed with conventional 0.55T reconstruction, and strong correlations (*R*^2^ ≥ 0.70) were only observed for the SER-based results. The angular discrepancy analysis also demonstrates that the estimated diffusion tensor orientations were generally more consistent between SER-based 0.55T and conventional 3T reconstruction results (median angular error = 11.1°) than they were between conventional 0.55T and conventional 3T reconstruction (median angular error = 15.3°).

**FIGURE 5:**
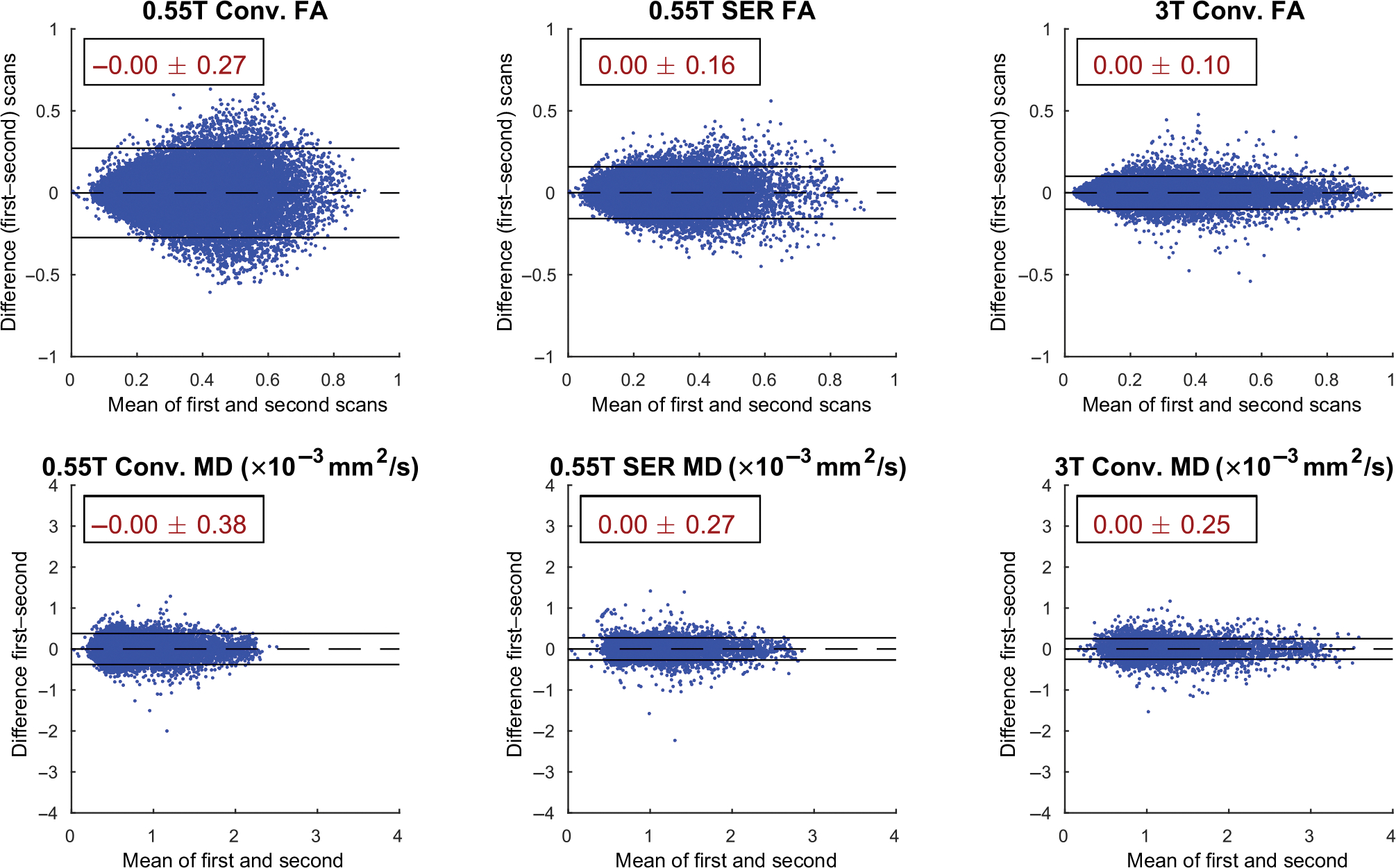
Bland–Altman plots evaluating the repeatability of (top) fractional anisotropy (FA) and (bottom) mean diffusivity (MD) values across repeated scans for (left) conventional 0.55T reconstruction, (center) signal-to-noise ratio-enhancing 0.55T reconstruction, and (right) conventional 3T reconstruction. Inset within each plot, we show the bias between the repeat scans (represented graphically with a dashed line) ± the 95% limits of agreement (represented graphically with solid lines). For visualization purposes, we are only showing 0.5% of the samples (randomly selected), but the statistics are calculated based on all of the samples.

Figure 5 shows the test–retest results. While all the methods show negligible bias, we observe that the FA and MD values obtained from 0.55T data using SER are more repeatable (smaller limits of agreement) than those obtained using conventional reconstruction of the 0.55T data. Interestingly, while the FA values were less repeatable using the 0.55T SER results than when using conventional reconstruction of the 3T data as expected, the repeatability of the MD values was similar for these two cases. The FA and MD values also showed reasonably good intra-subject coefficients of variation, which were less than 6% for FA and less than 7% for MD in white matter voxels, and less than 9% for FA and less than 5% for MD in nonwhite matter voxels for all cases.

## 4 | DISCUSSION AND CONCLUSIONS

Our results demonstrated that strong correlations (*R*^2^ ≥ 0.70) can be obtained between diffusion tensor imaging parametersobtainedfromtheinvivohumanbrainat0.55T and 3T, although the use of a denoising strategy can be important to mitigate SNR issues and improve repeatability at 0.55T. Overall, our results suggest that diffusion MRI is feasible using 0.55T MRI scanners, although the use of a higher-field scanner is likely still advantageous from an SNR perspective when available. While SNR was a significant limitation for our 0.55T diffusion MRI acquisition, it does have the advantage of reduced susceptibility-induced geometric distortion compared to 3T, in addition to the other potential advantages (e.g., value, accessibility, etc.) offered by lower-field scanners.

Although this work focused on one specific denoising strategy (that we believe has attractive features^21^), we expect that similar results would have been obtained with other denoising approaches.^*^ A detailed comparison between different denoising methods is beyond the scope of the present paper, although we refer interested readers to previous publications that include direct detailed comparisons of SER against competing approaches.^21–23,34^

A limitation of this study was that it only considered a small cohort of healthy subjects. The focus on healthy subjects prevents us from drawing conclusions about the behavior that might be observed in the presence of pathology, and the small sample size precludes more advanced statistical analyses. Nevertheless, the results are encouraging, and suggest that follow-up studies involving a larger subject cohort may be a valuable next step.

Although we observed strong correlations between the FA and MD values measured at 0.55 and 3T, we also observed some systematic differences. For example, the regression lines shown in Figure 4 had nonzero offsets and did not have slopes of 1. There are various possible explanations for these differences. First, it should be noted that relaxation times are different at 0.55 and 3T,^2^ and that parameter estimates obtained from biological tissues often represent a partial volume mixture of multiple microstructural tissue compartments that are each associated with distinct relaxation and diffusion parameters.^46^ This means that even if diffusion MRI data were acquired at the same echo time, the effects of relaxation are likely to cause different microstructural compartments to be more or less heavily weighted in the partial volume mixture at 0.55T than they were at 3T. In our study, this potential confound is exacerbated by the fact that we used different echo times at 0.55 and 3T. In addition, we also used different diffusion encoding methods to achieve the same b-value (i.e., the diffusion times and gradient strengths were likely different), which may also contribute to systematic differences in the estimated diffusion parameters. Finally, while we used an SNR-enhancement approach to mitigate noise at 0.55T,itisalsopossiblethatresidualnoiseeffectsmayhave contributed to systematic differences in the estimated diffusion tensor parameters. These differences may or may not be important for practical applications, as one could potentially use the estimated regression relationships to try and “convert” between 0.55 and 3T values if so desired.

## Supplementary Material

Supplementary Material

SUPPORTING INFORMATION

Additional supporting information may be found in the online version of the article at the publisher’s website.

**Figure S1.** Linear regression analyses between diffusion parameter values (FA and MD) obtained from 0.55T data and 3T data when the 0.55T data is denoised using MP-PCA. The formatting of these plots is identical to that of Figure 4.

## Footnotes

* For example, Figure S1 shows results suggesting that the MP-PCA denoising method^45^ also improves the correspondence between 0.55 and 3T results, albeit not as well as SNR-enhancing reconstruction did.
