# Supplementary material for: Diffusion tensor brain imaging at 0.55T: A feasibility study
Source: Magn Reson Med. Author manuscript; Available in PMC 2025 Dec 24. (PMC12732710; doi:10.1002/mrm.30156)
Supplement: Supplementary Material [file NIHMS2122415-supplement-Supplementary_Material.pdf]

Supporting Information

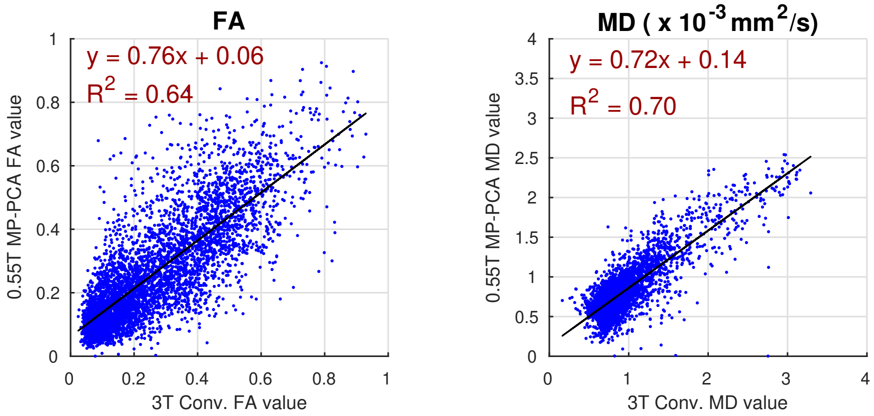

Supporting Information Figure S1: Linear regression analyses between diffusion parameter values (FA and MD) obtained from 0.55T data and 3T data when the 0.55T data is denoised using MP-PCA. The formatting of these plots is identical to that of Fig. 4.
